# Socio-Demographic Characteristics of COVID-19 Vaccine Recipients in Kwara State, North Central Nigeria

**DOI:** 10.3389/fpubh.2021.773998

**Published:** 2022-01-05

**Authors:** Ahmad Ibrahim Al-Mustapha, Musa Imam Abubakar, Muftau Oyewo, Rita Enyam Esighetti, Oluwaseun Adeolu Ogundijo, Lukman Dele Bolanle, Oluwatosin Enoch Fakayode, Abdullateef Saliman Olugbon, Michael Oguntoye, Nusirat Elelu

**Affiliations:** ^1^Department of Veterinary Services, Kwara State Ministry of Agriculture and Rural Development, Ilorin, Nigeria; ^2^Department of Veterinary Public Health and Preventive Medicine, Faculty of Veterinary Medicine, University of Ibadan, Ibadan, Nigeria; ^3^Infectious Diseases and One Health, Faculty of Pharmaceutical Sciences, Universite de Tours, Tours, France; ^4^Regional Disease Surveillance Systems Enhancement Project (REDISSE) II, Federal Ministry of Agriculture and Rural Development, Abuja, Nigeria; ^5^Nigerian Field Epidemiology and Laboratory Training Program, Abuja, Nigeria; ^6^Department of Public Health, Kwara State Ministry of Health, Ilorin, Nigeria; ^7^Kwara State Primary Healthcare Development Agency, Ilorin, Nigeria; ^8^Department of Veterinary Public Health and Preventive Medicine, Faculty of Veterinary Medicine, University of Ilorin, Ilorin, Nigeria

**Keywords:** COVID-19, vaccine acceptance, Nigeria, vaccine recipients, socio-demographic

## Abstract

Understanding key socio-demographic variables of 2019 coronavirus disease (COVID-19) vaccine recipients is crucial to improving its acceptance and Nigeria's COVID-19 control strategy. The survey was conducted as a non-probability cross-sectional survey of 2,936 COVID-19 vaccine recipients in Kwara State. Our findings revealed that 74% (*n* = 2,161) of the vaccine recipients were older than 40 years. Forty percent (*n* = 1,180) of the vaccine recipients earned a monthly income >100,000 Naira (equivalent to US $200). Most of the vaccine recipients (64%, *n* = 1,880) had tertiary education, while 15% (*n* = 440) of them had no formal education. Almost half of the recipients (47%, *n* = 1,262) were government employees and 28.8% (*n* = 846) of them had health-related backgrounds. Only 17% (*n* = 499) of the vaccine recipients have been screened for the severe acute respiratory syndrome coronavirus 2 (SARS-CoV-2), of which 21% (*n* = 105/499) of them were tested positive. Only 47% (*n* = 1,378) had been fully immunized. The prevalence of confirmed COVID-19 cases among COVID-19 vaccine recipients in Kwara State was 3.6% (*n* = 105/2,936). The most recurrent adverse events following immunization (AEFIs) among vaccine recipients were fever (14%, *n* = 411), pain at injection site (47%, *n* = 1,409), headache (19%, *n* = 558), and body weakness (9%, *n* = 264). The need to protect themselves from the deadly virus was the main reason that prompted people to voluntarily accept the COVID-19 vaccine. There is a high level of COVID-19 vaccine acceptance among respondents across all social classes including those with no formal education, those with very low monthly income (< US $2 per day), and in untested population. Hence, vaccine donors should prioritize equitable distribution to Low-and-Middle-income Countries (LMICs) such as Nigeria, and health authorities should improve vaccine advocacy to focus on vaccine safety and efficacy.

## Introduction

The 2019 coronavirus disease (COVID-19) has caused a serious global public health crisis. The pandemic also has severe socio-economic impacts resulting from international travel ban and movement restrictions (1). To curb its spread, several non-pharmaceutical interventions (such as the banning of international flights, use of nose masks, frequent handwashing, social distancing, and mandatory stay-at-home order) were instituted (1–3). However, the world needed vaccines to protect lives, restore the economy, and return to the “new normal” (4). Generally, vaccinations are safe and effective in boosting the host immunity before the host comes in contact with the wild-type pathogen (4).

As of August 31, 2021, more than 215 million confirmed COVID-19 cases and over 4.48 million deaths have been recorded in over 200 countries and some 4.6 billion doses of the COVID-19 vaccine have been administered (5, 6). Nigeria (with over 210 million inhabitants) has recorded 190,118 COVID-19 cases and 2,244 deaths from which Kwara State (with a population of 3.6 million) has recorded 3,513 confirmed COVID-19 cases (1.8% of Nigeria's COVID-19 cases) and 55 COVID-19-related deaths as of August 27, 2021 (7).

Nigeria received the first COVID-19 vaccine (4 million doses of the Oxford-AstraZeneca vaccine) in March 2021, out of which Kwara State received 55,790 doses of the vaccine in March and finished administering the vaccine in July 2021 (8, 9). This volume of vaccine is grossly inadequate for its 3.6 million inhabitants and might have very little impact in halting the spread of the severe acute respiratory syndrome coronavirus 2 (SARS-CoV-2) in the state.

The arrival of the second batch of COVID-19 vaccines (4 million doses of Moderna COVID-19 vaccine and 177,000 doses of the Johnson and Johnson COVID-19 vaccine) necessitates the urgent socio-demographic understanding of the COVID-19 vaccine recipients (10, 11). This will enable public health officials and policymakers to adopt the most effective health communication strategies necessary to increase the public acceptance of the COVID-19 vaccines.

Several studies have assessed and modeled the potential acceptance of the COVID-19 vaccines in Nigeria, Africa, and in some Low-and-Middle-income Countries (LMICs) (12–17). However, there is a dearth of information if these potential acceptance rates and campaigns translated actual acceptance of the vaccines.

Understanding the community-level vaccine acceptance dynamics is important to prevent a lag in vaccination in any cluster (community or zone); a condition that could prompt or promote the emergence and spread of vaccine-induced (resistant) variants (18, 19). This is the first study that provides a locally focused picture of COVID-19 vaccine recipients in Nigeria. We moved beyond potential vaccine acceptance rates to collecting and analyzing data aimed at understanding who took the vaccines and their reasons for accepting the vaccine.

Hence, this study presents the descriptive statistics of the COVID-19 vaccine recipients in Kwara State and provides information to guide public health decision-makers in Kwara state and the country at large.

## Methods

### Ethical Approval

The ethical clearance of this study was obtained from the Kwara State Ministry of Health, Ilorin, Nigeria (Reference number: MOH/KS/EHC/777/502). Written informed consent was obtained from each respondent after brief information on the purpose of the study was provided to them. The respondents either signed the paper questionnaire or ticked the box in the mobile application [open data kit (ODK)]. Participation in this survey was voluntary and without prejudice as specified in the World Medical Association Declaration of Helsinki Ethical principles (20).

### Study Design, Study Participants, and Sampling

This study was designed as a cross-sectional survey of adult respondents (18 years and above) from the three senatorial zones of Kwara State. The state has a human population of ~3.6 million people (21). The survey started on July 11 to 20, 2021. The study was conducted as a one-on-one interview at the designated COVID-19 vaccination centers across the state. Vaccination centers (*n* = 70) were strategically spread across the state (in each of the 16 local government areas of the state) by the Kwara State Primary Health Care Development Agency (Kwara PHCDA).

### Survey Methodology

The study was administered as one-on-one interviews to vaccine recipients from selected COVID-19 vaccination centers across the state using a multi-stage sampling technique (Supplementary Table 1). Three local government authorities (LGAs) were selected by simple random sampling technique from each senatorial zone of the state (a total of 9 LGAs). Furthermore, three (3) COVID-19 vaccination centers were selected from each of the LGAs. Data collectors were trained on the methodology of the survey and were sent to pre-selected vaccination centers in their respective LGA. Each respondent was interviewed independently to prevent clustering of responses. The questionnaire (in English or verbally translated to the respondents' languages where necessary) was administered while the vaccine recipients waited to be registered or inoculated with the vaccine. Each vaccine recipient was selected by systematic random sampling with a sampling frame of two. Hence, we sampled the first, fourth, seventh person and so on. However, in certain vaccination centers with few vaccine recipients (<10), we sampled all of them.

With an estimated population of 3.6 million people in the state, at a 96% confidence interval, assuming a 50% vaccine acceptance rate, and a 4% margin of error, the required sample size is 849 vaccine recipients per senatorial zone. Hence, we calculated at least 2,547 individuals were needed. Eventually, the survey instrument was administered to 2,936 COVID-19 vaccine recipients across the three senatorial zones (9 LGAs and 27 vaccination centers) of the state. Of these, 1,174 (40%) were from Kwara Central, while other respondents were equally distributed between Kwara North and Kwara South (881 respondents, respectively).

### Questionnaire Design

A structured questionnaire was developed for this study and administered as paper questionnaires or through the ODKs on mobile phones to obtain information from vaccine recipients. The choice of both paper and ODK questionnaire was based on the availability of android phones to some data collectors. Two independent reviewers were selected to validate the questionnaire to assess the content validity, clarity, ease of response, scope, and the face validity of the questions. Furthermore, the Cronbach alpha test was used to assess the reliability of the survey instrument (with a score of 0.7). Finally, to check for technical glitches, the aptness of the survey tool, and typographical errors, a pre-test of the survey was administered to 10 vaccine recipients from each of the 27 selected vaccination centers. Responses obtained from the pre-test were not incorporated in the final analysis of the data. The survey instrument had only two sections: A and B. Section A assessed the demographic characteristics of the respondents (age, gender, level of education, monthly income, occupation, background, and marital status), while Section B focused on the COVID-19 vaccine (Supplementary File 1).

### Data Analysis

The data were summarized using Microsoft Excel (Microsoft, Redmond, WA, USA) and subjected to further statistical analysis using Minitab v.17 (Pennsylvania, USA). Qualitative data were presented as frequencies and proportions.

## Results

### Demographics of COVID-19 Vaccine Recipients in Kwara State

Our findings revealed that all age groups received the vaccine. However, persons older than 40 years represented 74% of the total respondents. Forty percent (*n* = 1,180) of the vaccine recipients earned a monthly income >100,000 Naira (equivalent to US $200). Most of the vaccine recipients (64%, *n* = 1,880) had tertiary education, while 15% (*n* = 440) of them had no formal education (Table 1). Almost half of the recipients (47%, *n* = 1,262) were government employees from the civil service, academia, government teachers, military, para-military, and retirees. Only 846 (28.8) of the vaccine recipients had health-related backgrounds (medical sciences, medical laboratory scientists, community health workers, nurses, etc.). Most of the vaccine recipients (80%, *n* = 2,349) were married.

**Table 1:** Socio-demographic profiles of 2019 coronavirus disease (COVID-19) vaccine recipients in Kwara State (*n* = 2,936).

**Variable**	**Frequency (%)**
**Age (Years)**	
18–29	352 (12)
30–39	423 (14.4)
40–49	752 (25.6)
50–59	681 (23.2)
>60	728 (24.8)
**Gender**	
Female	1,174 (40)
Male	1,762 (60)
**Monthly income**	
30,000 or less	629 (21.4)
30,000 – 100,000	1,127 (38.4)
>100,000	1,180 (40.2)
**Level of education**	
No formal education	440 (15)
Primary education	264 (9)
Secondary education	352 (12)
Tertiary education	1,880 (64)
**Occupation**	
Government employee	1,262 (43)
Private company or self-employed	1,673 (57)
**Background**	
Health-related	846 (28.8)
Non-health related	2,090 (71.2)
**Marital status**	
Single	446 (15.2)
Married	2,349 (80)
Divorced/Widowed	141 (4.8)

Our survey showed that only 499 (17%) of the vaccine recipients have been screened for the SARS-CoV-2. Of the 499 persons previously screened for the SARS-COV-2, 105 (21%) of them were tested positive for SARS-CoV-2 (Table 2). Hence, the prevalence of confirmed COVID-19 cases among COVID-19 vaccine recipients in Kwara State was 3.6% (*n* = 105/2,936). Of the 2,936 vaccine recipients included in this survey, only 1,378 had been fully immunized (taken the two doses of the Oxford-AstraZeneca vaccine). The most recurrent adverse event following immunization (AEFI) among vaccine recipients (from second dose recipients only) were fever (30%, *n* = 411), pain at injection site (87%, *n* = 1,198), headache (40%, *n* = 558), and body weakness (19%, *n* = 264). The need to protect themselves from the deadly virus was the main reason that prompted people to voluntarily accept the COVID-19 vaccine.

**Table 2:** Responses related to COVID-19 vaccine in Kwara State (*n* = 2,936).

**Variable**	**Frequency (%)**
1a. Prior screening for the SARS-CoV-2?	
No	2,437 (83)
Yes	499 (17)
1b. Result of SARS-CoV-2 screening test*	
No	394 (79)
Yes	105 (21)
2. Dose of vaccine	
1^st^ dose	1,558 (53)
2^nd^ dose	1,378 (47)
3. Known underlying condition	
No	2,730 (93%)
Yes	206 (7%)
4. Adverse events following your inoculation with the COVID-19 vaccine	
No	843 (28.7)
Yes	2,093 (71.3)

More vaccine recipients have been screened in Kwara Central than in the other two senatorial zones. However, there were no statistically significant differences in the demographics of the vaccine recipients from the three senatorial zones. Most vaccine recipients (89%, *n* = 2,613/2,936) across the state voluntarily accepted the vaccine because of their need to protect themselves as well as others around them.

## Discussion

Vaccinations are one of the greatest public health interventions against infectious diseases (22). Hence, the acceptance of the COVID-19 vaccine is as important as its availability and equitable distribution, especially in resource-limited settings. Our study contributes to the emerging picture on COVID-19 vaccine acceptance by focusing on data obtained from 2,936 vaccine recipients across 45 vaccination centers across Kwara State.

There are five main individual determinants for vaccine acceptance: confidence, complacency, convenience, risk calculation, and collective responsibility (23, 24). These determinants have made Nigerians accept the COVID-19 vaccines despite concerns about vaccine safety and efficacy especially with the rapid pace of COVID-19 vaccine development, reports of thrombosis associated with the Oxford-AstraZeneca COVID-19 vaccine, consistently low COVID-19 cases and deaths in Nigeria, poor perception of COVID-19 severity, mistrust in government's handling of the pandemic, lack of economic palliatives for citizens, and poor healthcare infrastructure (25, 26).

Consistent with previous findings, our findings revealed higher vaccine recipients among men than women (15, 27). We advocate that community engagement activities involving social learning and women groups could help increase vaccination acceptance among women using the Social and Behavioral Change Communication (SBCC) approach. This will persuade more women to accept the vaccine.

Although more than half of the vaccine recipients (*n* = 1,880) had tertiary education, our findings showed that about a quarter of the recipients (*n* = 704) had no formal education or just went through basic school. In the same vein, 21% of the vaccine recipients included in this study earned the minimum wage (monthly income of 30,000 Naira or US $59) also voluntarily accepted the vaccine. It is encouraging to know that these sub-populations voluntarily presented themselves for the COVID-19 inoculation. So, we believe that the general public will adopt and comply with the non-pharmaceutical intervention guidelines (frequent handwashing, use of face masks, social distancing, cough etiquette, etc.) if the government re-strategize its mass COVID-19 advocacy campaigns.

There were no statistically significant differences in the demographics of the vaccine recipients from the three senatorial zones. However, more persons have been screened for SARS-CoV-2 in Kwara Central than in the other two zones. This could be because the only COVID-19 testing center is located in Kwara Central. However, there are designated sample collection points in every local government of the state. Hence, the health authorities should provide at least a COVID-19 screening facility in each of the senatorial zones.

Consistent with other reports, Nigeria (Kwara State included) must improve its COVID-19 testing capacity (including molecular genomic surveillance) (25, 28, 29). This poor COVID-19 testing mechanism is evident in that of the 2,936 vaccine recipients included in this study, only 499 (17%) of them have been tested for the SARS-CoV-2. The poor testing rate could be due to the stigma associated with COVID-19 especially at the rural level []. This has made people shy away from testing. However, with the arrival of the vaccine (the anticipated cure), people voluntarily presented themselves for inoculation even at the grass-root level.

With a prevalence of 3.6% in the tested sub-population, health agencies must ensure that statewide or nationwide screening for SARS-CoV-2 is intensified to determine the true prevalence of COVID-19. This will help identify positive predictors, predisposing factors, and clusters of infections across the state and the country (25).

The self-reported AEFI recorded in the 1,378 fully immunized persons were transient side effects (pain at the injection site, headache, fever, fatigue, etc.). This is consistent with several reports that COVID-19 vaccines were safe (30–34). Contrary to the wildly circulated magnetic theory of the COVID-19 vaccine (35, 36), none of the recipients reported being magnetic. This shows that the vaccine is safe and effective to reduce hospitalizations and the tendency of spreading the disease (15–17).

Another major focus of this study was to understand what prompted vaccine recipients to voluntarily accept the vaccine despite long queues and hassles in the vaccination centers. We found that the need to protect oneself from deadly diseases and prevent complications of underlying disease conditions (co-morbidities) were the most prominent reasons for accepting the COVID-19 vaccine among our study participants. This is similar to the report of Solis-Arce et al. (27).

To further boost the vaccination acceptance among Nigerians, health agencies should adopt the WHO's Behavioral and Social Drivers of Vaccination model (BeSD) which suggests that countries should reduce access barriers to COVID vaccinations (37). Dependent on the availability of vaccines, health authorities should make vaccines closer to the populace by increasing vaccination centers across the state, reducing the time lag in the online registration of recipients, and designing integrated health programs that will leverage on the vaccination centers' workforce and facilities.

The implications of our findings for public health were that there is a high level of vaccine acceptance among respondents with no formal educations, those with very low monthly income (< US $2 per day), and in the untested population than initially anticipated. This should encourage vaccine donors to prioritize equitable distribution to LMICs such as Nigeria as with the constantly increasing population of LMICs, the world could in a short time attain herd immunity (27).

The main strength of this study is its large sample size, the timeliness of the study before the second phase of the COVID-19 vaccination in Nigeria, and its widespread participants across the study area. However, the main limitation is that our data might not be representative of all COVID-19 vaccine recipients in the state (*n* = 57,790).

In Nigeria, an increased vaccine acceptance rate can be achieved by persuading the populace through community engagement activities and employing SBCC strategies. We advocate that vaccine messaging should focus on vaccine safety, efficacy, and the lack of side effects even in persons with co-morbidities. While some studies have reported that cash tokens or in-kind incentives have increased vaccination rate especially in childhood immunization programs in several LMICs (37–41), this study observes a positive attitude of vaccine recipients, and we opine that vaccination date reminders and few logistic improvements would suffice and no cash incentives are needed yet.

## Conclusion

This study provided socio-demographic information on COVID-19 vaccine recipients across Kwara State. There was high vaccination acceptance among all social classes. Increased vaccine acceptance can be achieved using the community engagement approach with an emphasis on vaccine safety and efficacy.

## Data Availability Statement

The raw data supporting the conclusions of this article will be made available by the authors, without undue reservation.

## Ethics Statement

The studies involving human participants were reviewed and approved by Ethical Review Board of the Kwara State Ministry of Health, Ilorin, Nigeria (Reference number: MOH/KS/EHC/777/502). The participants provided their written informed consent to participate in this study.

## Author Contributions

All authors contributed equally to the conception, analysis, and writing of the manuscript.

## Conflict of Interest

The authors declare that the research was conducted in the absence of any commercial or financial relationships that could be construed as a potential conflict of interest.

## Publisher's Note

All claims expressed in this article are solely those of the authors and do not necessarily represent those of their affiliated organizations, or those of the publisher, the editors and the reviewers. Any product that may be evaluated in this article, or claim that may be made by its manufacturer, is not guaranteed or endorsed by the publisher.
