# Supplementary material for: Socio-Demographic Characteristics of COVID-19 Vaccine Recipients in Kwara State, North Central Nigeria
Source: Front Public Health. 2022 Jan 5;9:773998. doi: 10.3389/fpubh.2021.773998 (PMC8766999; doi:10.3389/fpubh.2021.773998)

Supplementary data

Figure S1. Flow-diagram for multi-stage sampling of COVID-19 recipients in Kwara State (n=2,936).

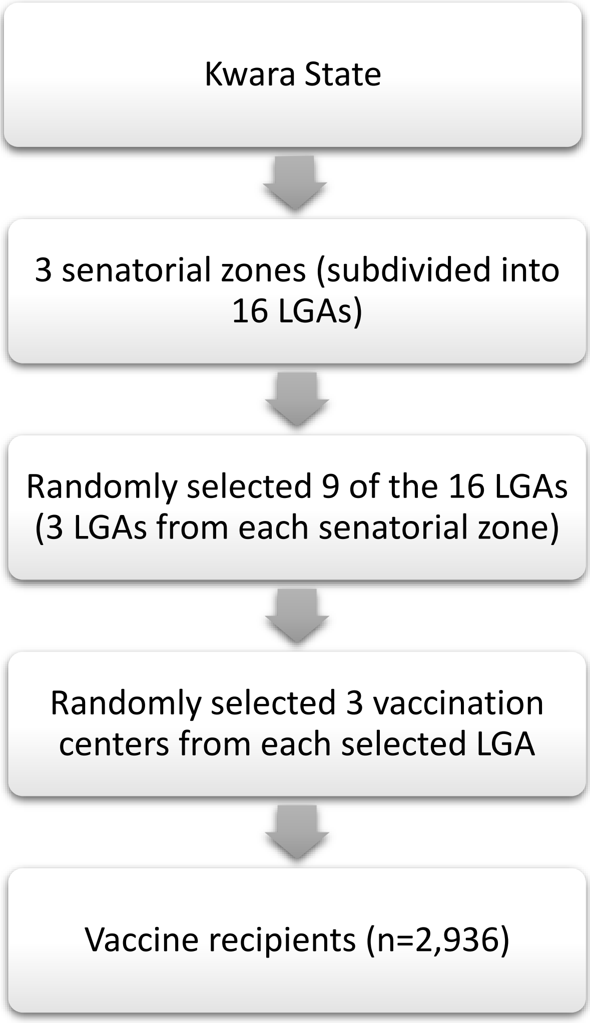

Supplement: Supplementary file 1 [file Data_Sheet_1.pdf]
